# Catheter ablation versus medical rate control for persistent atrial fibrillation in patients with heart failure: A PRISMA-compliant systematic review and meta-analysis of randomized controlled trials

**DOI:** 10.1097/MD.0000000000004377

**Published:** 2016-07-29

**Authors:** Min Zhu, Xinbin Zhou, Hongwen Cai, Zhijun Wang, Huimin Xu, Shenjie Chen, Jie Chen, Xiaoming Xu, Haibin Xu, Wei Mao

**Affiliations:** a Department of Cardiology, First Affiliated Hospital of Zhejiang Chinese Medical University; b Department of Pharmacy, Second Affiliated Hospital, Zhejiang University School of Medicine, Hangzhou, Zhejiang, China.

**Keywords:** atrial fibrillation, catheter ablation, heart failure, medical rate control, systematic review

## Abstract

**Background:**

The effectiveness of restoring the sinus rhythm by catheter ablation relative to that of medical rate control for persistent atrial fibrillation (AF) patients with heart failure (HF) remains to be defined.

**Methods:**

We systematically searched Embase, Pubmed, the Cochrane Library, and ClinicalTrials.gov for articles that compared the outcomes of interest between catheter ablation and medical rate control therapy in persistent AF patients with HF and left ventricular systolic dysfunction (LVSD). The primary endpoint was the change in the left ventricular ejection fraction (LVEF) following catheter ablation or medical rate control therapy relative to baseline. Other endpoints included changes in cardiac function and exercise capacity, including the New York Heart Association (NYHA) class, the brain natriuretic peptide (BNP) level, the peak oxygen consumption (peak VO_2_), the 6-minute walk test (6MWT) results, and quality of life (QOL).

**Results:**

Three randomized controlled trials (RCTs) with 143 patients were included. At the overall term follow-up, catheter ablation significantly improved the LVEF (mean difference [MD]: 6.22%; 95% confidence interval [CI]: 0.7–11.74, *P* = 0.03) and peak VO_2_ (MD: 2.81 mL/kg/min; 95% CI: 0.78–4.85, *P* = 0.007) and reduced the NYHA class (MD: 0.9; 95% CI: 0.59–1.21, *P* < 0.001) and the Minnesota Living with Heart Failure Questionnaires (MLHFQ) scores (MD: −11.05; 95% CI: −19.45 — −2.66, *P* = 0.01) compared with the medical rate control for persistent AF patients with HF. Alterations in parameters, such as the BNP level, 6MWT, and Short Form-36 (SF-36) questionnaire scores also revealed trends that favored catheter ablation therapy, although these differences were not significant.

**Conclusion:**

Catheter ablation resulted in improved LVEF, cardiac function, exercise capacity, and QOL for persistent AF patients with HF compared with the medical rate control strategy.

## 1 Introduction

Atrial fibrillation (AF) and heart failure (HF) are 2 rapidly expanding cardiac diseases that commonly coexist and adversely aggravate each other. Optimal therapeutic approaches that target AF and HF require further exploration.

Restoring the sinus rhythm seems to improve the outcomes of AF patients with HF; however, several studies have demonstrated that pharmacologic rhythm control strategies do not improve outcomes regardless of whether they are applied to AF patients with^[1]^ or without^[2]^ HF. This lack of effect may be due to the difficulty of maintaining the sinus rhythm via pharmacologic approaches alone and the adverse effects of antiarrhythmic drugs that negate the benefits of the sinus rhythm. Therefore, the restoration of the sinus rhythm via catheter ablation in these patients has attracted attention and has been proved to be effective compared with medical rhythm control therapy, but only in AF patients with normal heart function.^[3–5]^

A recent meta-analysis that examined the effects of catheter ablation in AF and HF patients revealed that catheter ablation resulted in significant improvements in left ventricular function. However, because this was a single-arm analysis with the inclusion of varied control groups and significant heterogeneity (*I*^2^ = 92.9%), the results of this study should be interpreted with caution.^[6]^

In recent years, the treatment for AF and HF patients has shifted from rhythm to rate control, and medical rate control is even recommended as the 1st-line therapy for such patients in the acute phase.^[7]^ However, the role and benefit of this treatment have been challenged, for example, a recent study demonstrated that a slowed ventricular rate was not associated with better outcomes for chronic HF patients with AF,^[8]^ and a recent meta-analysis also indicated no significant beneficial effect of beta blockers for these patients.^[9]^

Several studies have investigated whether the restoration of the sinus rhythm via catheter ablation improves the outcomes of AF patients with HF.^[10–15]^ However, these studies have reported inconsistent results, and few of them were randomized; therefore, the efficacy of catheter ablation relative to medical rate control remains to be determined.

Therefore, we performed a systematic review and meta-analysis of studies to discuss the efficacy and safety of restoring the sinus rhythm using catheter ablation in AF patients with HF compared with the efficacy and safety of pharmacological rate control therapy.

## 2 Materials and methods

This study was performed following the Preferred Reporting Items for Systematic Reviews and Meta-Analyses (PRISMA) guidelines.^[16]^ As all analyses were based on previous published studies, no ethical approval and patient written informed consent were required.

### 2.1 Search strategy and selection criteria

We systematically searched Embase, Pubmed, the Cochrane Library, and ClinicalTrials.gov for articles until December 20, 2015, using the following terms and variants thereof: (“atrial fibrillation” OR “persistent atrial fibrillation”) AND (“heart failure” OR “left ventricular systolic dysfunction” OR “reduced left ventricular systolic function”) AND (ablation OR “catheter ablation” OR “pulmonary vein isolation”). Additionally, we manually searched the references of the selected articles, relevant reviews, and previous meta-analyses for potentially relevant citations. Only randomized controlled trials (RCTs) in the English language were included, but no publication status restriction was imposed.

For inclusion in our research, the studies were required to meet the following criteria: RCT; original data regarding catheter ablation versus a medical rate control strategy for persistent AF patients with HF were included; more than 10 patients included; the length of follow-up was at least 6 months; and the outcomes of interest were included. The exclusion criteria were as follows: not published in English; AF with diastolic HF or diastolic dysfunction; restoration of the sinus rhythm by surgical ablation, e.g., the Maze-III procedure; and rate control via atrioventricular node ablation.

### 2.2 Data collection and quality assessment

The data extraction and quality assessment were performed independently by 2 reviewers, and disagreements were resolved by consensus. The following data were extracted: study design, number of patients assigned to each group, participant characteristics, details of the ablation procedure and medical therapy, duration of follow-up, and outcomes of interest.

The quality of RCTs was assessed by Cochrane Collaboration tool (Cochrane handbook for systematic reviews of interventions).^[17]^

### 2.3 Outcomes

The primary outcome of interest was the change in the left ventricular ejection fraction (LVEF) following catheter ablation or medical rate control therapy relative to baseline. Secondary outcomes included changes in cardiac function, exercise capacity, and quality of life (QOL). Cardiac function and exercise capacity were assessed via the New York Heart Association Class (NYHA class), the brain natriuretic peptide (BNP) level, the peak oxygen consumption (peak VO_2_), and the 6-minute walk test (6MWT) distance. QOL was assessed with the Short Form-36 (SF-36), Minnesota Living with Heart Failure Questionnaire (MLHFQ), and the Kansas City Cardiomyopathy Questionnaire (KCCQ).

### 2.4 Statistical analysis

We used the weighted mean differences and the 95% confidence intervals (CIs) for the continuous variables. Heterogeneity was assessed with the *I*^2^ statistic, and <25% was considered indicative of low heterogeneity. When heterogeneity was present, we used a random effects model and sought to identify the source (s) of the heterogeneity, otherwise a fixed effects model was applied. Publication bias was graphically analyzed with funnel plots. We also applied Egger and Begger statistical tests. A *P* value of <0.05 was considered statistically significant.

When analyzing the outcomes, when a relevant study did not directly provide certain outcome change data, we then set a correlation coefficient equal to the average of the remaining studies for that study to calculate the target change from baseline. In this condition, sensitivity analyses were also performed using several different correlation coefficients ranging from 0 to 1.

Analyses were performed using Review Manager version 5.3 (The Cochrane Collaboration, Copenhagen, Denmark), while Egger and Begger tests were performed using STATA version 12.0 (STATA Corporation, TX).

## 3 Results

### 3.1 Eligible studies and characteristics

**Figure 1:**
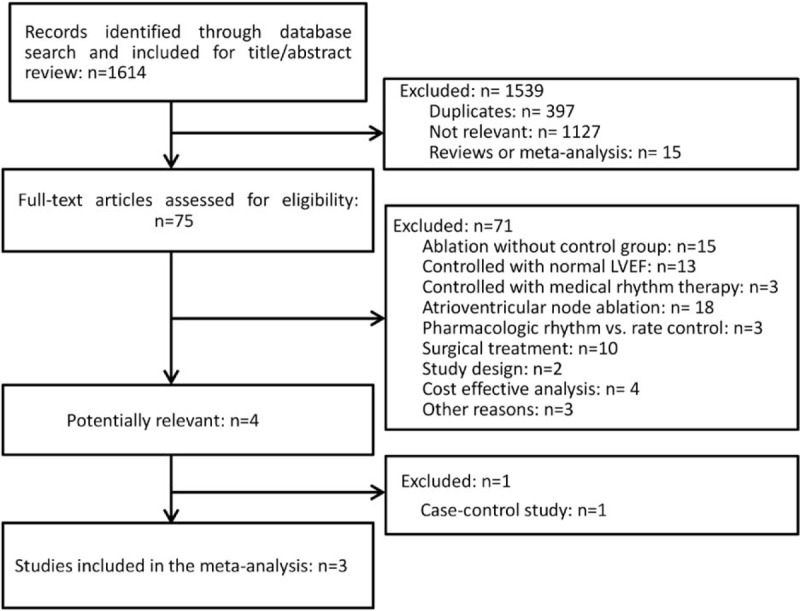
Flow chart of the systematic literature research for the meta-analysis.

In the initial research, we identified a total of 1614 studies of which 75 studies were potentially relevant and the full texts of these studies were further assessed. Ultimately, only 3 studies with a total of 143 patients were included in the analysis.^[18–20]^ Moreover, no additional studies were found when we manually searched the references of the selected articles, relevant reviews, and meta-analyses (Fig. 1).

All the included studies were designed to compare catheter ablation with medical rate control therapy in persistent AF patients with HF. The baseline characteristics of the included studies are presented in Table 1. Briefly, the mean LVEFs ranged from 24% to 39.3%, and the sample sizes ranged from 41 to 52. The mean age of the patients ranged from 57 to 63 years old, and the length of follow-up ranged from 6 to 12 months.

**Table 1:**
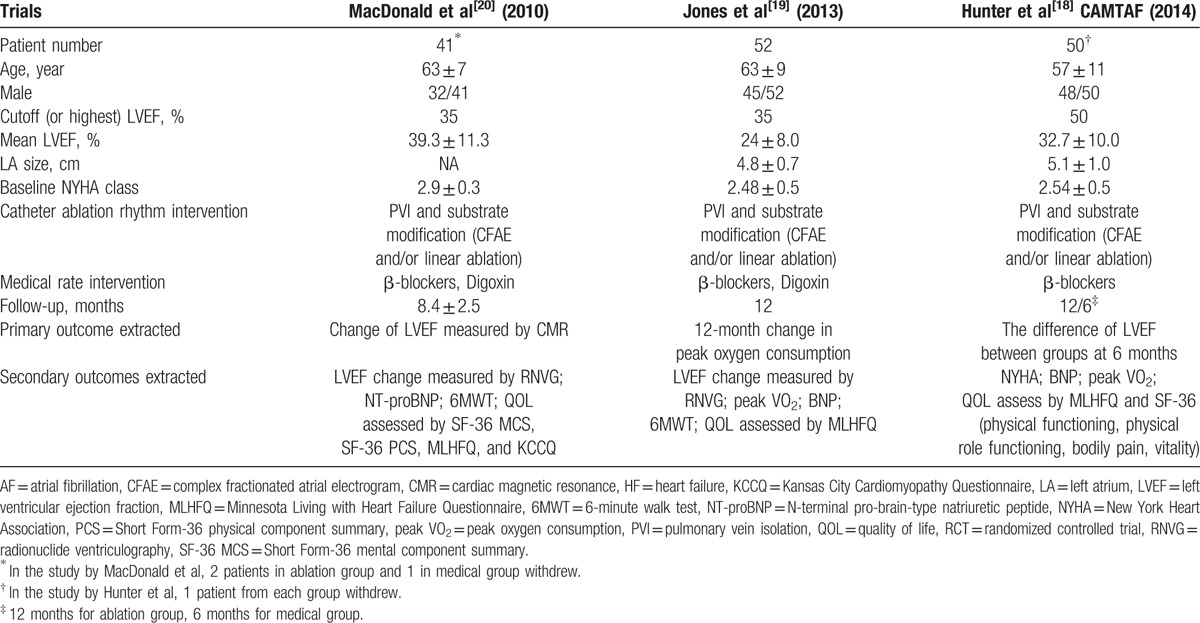
Trials’ main characteristics.

The LVEF was measured via echocardiography in 1 study,^[18]^ and via radionuclide ventriculography (RNVG)^[19]^ or both RNVG and cardiovascular magnetic resonance (CMR)^[20]^ in the remaining 2 studies.

In the study by MacDonald et al,^[20]^ the LVEF change measured by CMR was the primary end point and that measured by RNVG was a secondary end point. Therefore, we selected the LVEF values as assessed by CMR rather than RNVG for the pooling analysis.

The mean durations of AF before inclusion varied between studies with ablation arms ranging from 24 to 44 months and medical arms ranging from 23 to 64 months. Moreover, the underlying heart disease varied with the ischemic etiology ranged from 23% to 50% in the ablation arm and from 27% to 47% in the rate control arm (*P* > 0.05). The dilated cardiomyopathies ranged from 14% to 31% and from 29% to 32% (*P* > 0.05) in the ablation and rate control arms, respectively.

The catheter ablation procedure was pulmonary vein isolation combined with substrate modification (complex fractionated atrial electrogram and/or linear ablation), and the patients’ heart rates were controlled with β-blockers^[18]^ or β-blockers combined with digoxin.^[19,20]^

**Figure 2:**
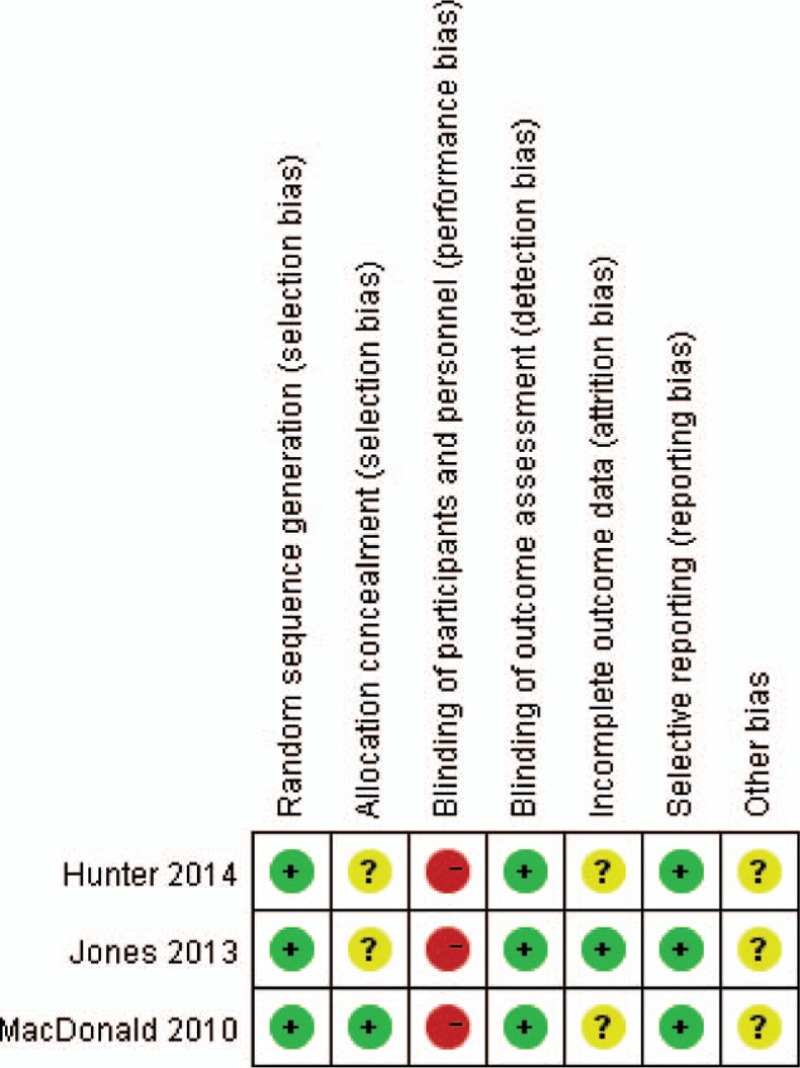
Quality assessments of the randomized controlled trials (RCTs) with the Cochrane Collaboration tool.

All 3 of the RCTs included in our analysis had relatively low risks of bias according to the Cochrane Collaboration tool (Fig. 2). The nature of the interventions did not permit blinding, but the assessors of the follow-up data measurements were blinded.

In cases with such low numbers of available studies, it is recommended that only random effects models should be used due to the fear that significant results based on a fixed effects model could be nonsignificant.

### 3.2 Primary end point

#### 3.2.1 Left ventricular ejection fraction

Among all the studies, mean baseline LVEF ranged from 15.1% to 31.8% in the ablation group and 19.6% to 33.7% in the medical rate control group. One^[18]^ of the 3 studies reported significant LVEF improvement, whereas the remaining studies^[19,20]^ did not.

**Figure 3:**
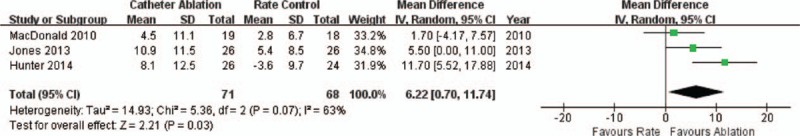
Forest plot comparing catheter ablation and medical rate control in terms of LVEF changes in patients with AF and HF. AF = atrial fibrillation, HF = heart failure, LVEF = left ventricular ejection fraction.

The pooled analysis indicated that, compared with medical rate control therapy, the restoration of the sinus rhythm by catheter ablation resulted in a significant improvement in the LVEF with a mean difference (MD) of 6.22% (95% CI: 0.7–11.74, *P* = 0.03; Fig. 3).

**Figure 4:**
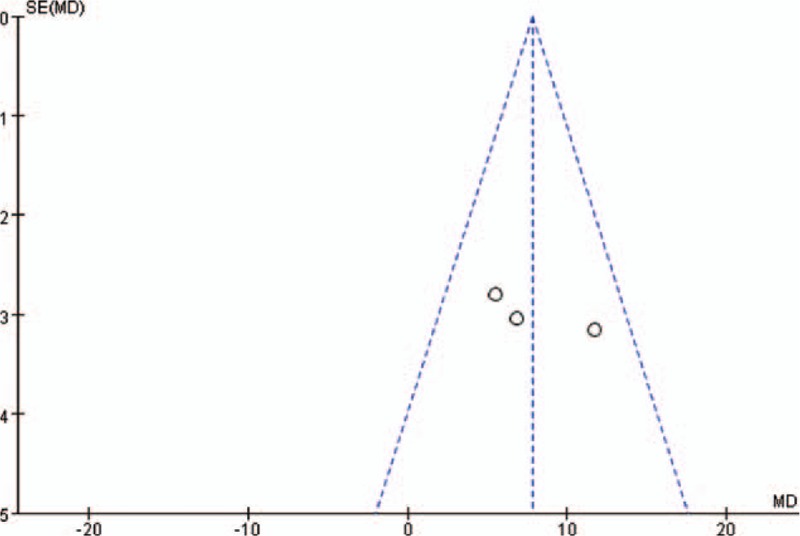
Funnel plot of the studies included in the meta-analysis.

No significant publication bias was found in the funnel plot (Fig. 4) or revealed by the Egger and Begger tests (Egger: *P* = 0.336; Begger: *P* = 0.296). However, moderate heterogeneity among the studies was detected (*I*^2^ = 63%, *P* = 0.07).

As we chose only LVEF assessed by CMR from the study of MacDonald,^[20]^ we next examined whether there was any change if the RNVG was selected from this study for the pooled analysis and found that results were not altered; the MD of the LVEF changed to 7.81% (95% CI: 4.17–11.45, *P* < 0.001). Moreover, no obvious heterogeneity was detected (*I*^2^ = 14%, *P* = 0.31).

### 3.3 Secondary end points

#### 3.3.1 Cardiac function and exercise capacity

**Figure 5:**
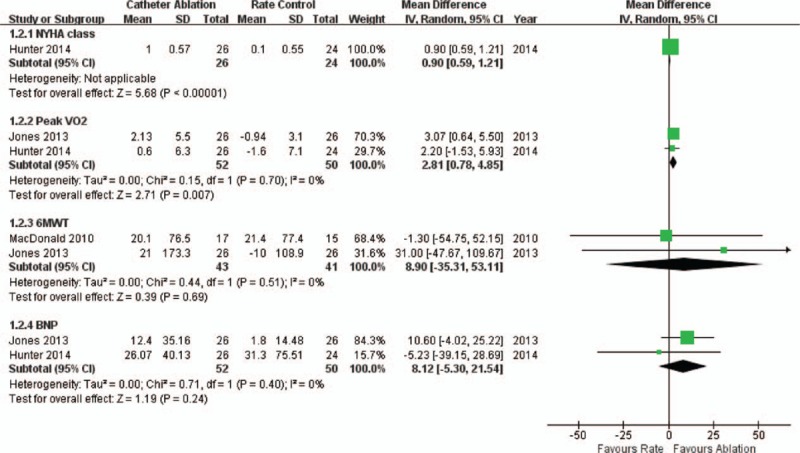
Forest plot comparing catheter ablation and medical rate control in terms of the cardiac functions and exercise capacities of patients with AF and HF. AF = atrial fibrillation, HF = heart failure.

Two of the studies^[18,19]^ reported baseline mean NYHA class grades of 2.46 and 2.6 in the ablation arm and 2.5 and 2.5 in the rate control arm. However, only the study of Hunter et al^[18]^ provided data about the NYHA class alterations relative to baseline. At 6 months of follow-up, the patients in ablation group achieved a significant improvement (reduction) in their NYHA score compared with the patients in the rate control group (MD: 0.9; 95% CI: 0.59–1.21, *P* < 0.001; Fig. 5). In the ablation arm, this NYHA class improvement was also maintained at the 1 year follow-up (1.7, CI: 1.4–2.0).

The studies of both Jones^[19]^ and Hunter^[18]^ observed reduction in the BNP levels in the ablation groups. In the study of Jones,^[19]^ the BNP level in the ablation arm exhibited a significant decrease of −124 (−284–0) pg/mL compared with the rate control arm (−18 (−86–+31) pg/mL) at both 6 (*P* = 0.038) and 12 months (*P* = 0.045). Moreover, in the study by Hunter,^[18]^ the BNP levels significantly differed between the 2 arms (126 pg/mL [CI: 63–189] in the ablation arm versus 327 pg/mL [CI: 172–481] in the rate control arm) at the 6-month follow-up (*P* = 0.014); however, the decreases compared to baseline failed to significantly vary between the 2 groups. Moreover, the pooled analysis revealed no significant difference in the BNP reduction between the 2 arms (MD: 81.2 pg/mL; 95% CI: −53.0–215.4, *P* = 0.24; Fig. 5).

Changes in the N-terminal pro-brain-type natriuretic peptide (NT-proBNP) were also assessed in the study by MacDonald et al,^[20]^ but no significant difference was detected between the 2 groups (−196 ± 1469 vs +85 ± 648 pg/mL, *P* = 0.45).

Two studies provided data about the peak VO_2_ changes. In the study by Jones et al,^[19]^ which used this parameter as the primary endpoint, the peak VO_2_ significantly increased by 2.13 (−0.10 to 4.36) mL/kg/min in the ablation group compared with a reduction of 0.94 (−2.21 to 0.32) mL/kg/min in the rate control group at a 12-month follow-up (*P* = 0.018). The study by Hunter et al^[18]^ also reported a significant peak VO_2_ difference between the 2 arms (22.4 [CI: 19.7–25.1] vs 17.7 [CI: 15.0–20.4]) mL/kg/min, *P* = 0.014) at 6 months. A pooled analysis detected a significant improvement in the peak VO_2_ in the ablation arm compared with that in the rate control arm (MD: 2.81 mL/kg/min; 95% CI: 0.78–4.85, *P* = 0.007; Fig. 5).

The 6MWT was performed in 2 of the studies. In the study by MacDonald,^[20]^ there was no significant difference between the ablation and rate control arms, which exhibited 6MWT distance increases of 20.1 ± 76.5 and 21.4 ± 77.4 m, respectively (*P* = 0.96). In the study by Jones,^[19]^ the 6MWT distance increased in the ablation arm (+21 m, −51–+89 m) and decreased in the rate control arm (−10 m, −73–+15 m) at 12 months, but this difference was not significant (*P* = 0.095). A pooled analysis also revealed no significance between the 2 arms (*P* = 0.69, Fig. 5).

#### 3.3.2 Quality of life

**Figure 6:**
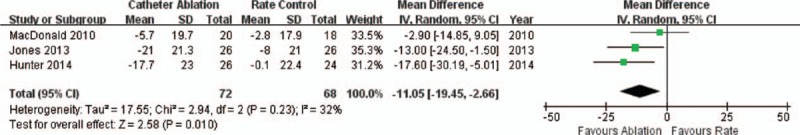
Forest plot comparing catheter ablation and medical rate control in terms of MLHFQ score changes in the patients with AF and HF. AF = atrial fibrillation, HF = heart failure, MLHFQ = Minnesota Living with Heart Failure Questionnaire.

All studies contributed to the analysis of the MLHFQ. A significant improvement (score reduction) in MLHFQ was detected in the patients who were treated with ablation with an MD of 11.05 in the score reduction (95% CI: −19.45 — −2.66, *P* = 0.01) compared to rate control therapy. However, some heterogeneity was detected (*I*^2^ = 32%, *P* = 0.23; Fig. 6).

As 1 study did not directly provide the MDs and standard deviations of the MLHFQ score changes, we set the correlation coefficient equal to the average of the other 2 studies, that is, 0.5. In a sensitivity analysis, the setting of the correlation coefficient to 0, 0.3, 0.7, or 1 did not change the pooled results, which exhibited a mean score difference of 11.05 and a *P* value < 0.05 in all circumstances.

The SF-36 was applied in 2 studies. In the study by Hunter et al,^[18]^ several aspects of the SF-36 questionnaire exhibited significant improvements in the ablation arm compared to the rate control arm that included physical functioning (71% in the ablation group vs 49.1% in the rate control group, *P* = 0.007), physical role functioning (67.5% vs 42.4%, *P* = 0.004), bodily pain (78.8% vs 57.1%, *P* = 0.005), and vitality (54.3% vs 36.4%, *P* = 0.009). These differences led to a significant improvement in QOL. Similarly, in the study by MacDonald et al,^[20]^ the SF-36 physical component summary score exhibited a significant increase of 4 ± 9.5 in the ablation arm compared with a reduction of 1 ± 4.4 in the rate control arm (*P* = 0.042), but the SF-36 mental component summary score failed to exhibit a significant difference between 2 arms and neither did another assessment of the KCCQ in this study.

## 4 Discussion

In the present article, we comprehensively reviewed and pooled results from 3 RCTs with a total of 143 patients who compared catheter ablation and medical rate control therapy for persistent AF patients with HF in terms of LVEF, cardiac function, exercise capacity, and QOL.

The results revealed that for persistent AF patients with HF, catheter ablation resulted in a significant improvement in the LVEF compared with pharmacological rate control therapy with an MD of 6.22%. Moderate heterogeneity was detected.

When we sought to identify the source (s) of this heterogeneity, we found that if the LVEF change as measured by RNVG in the study of MacDonald^[20]^ was selected, no obvious heterogeneity was detected, which indicated that the heterogeneity may have resulted from the 3 different LVEF assessment measures that were applied, that is, CMR in the study by MacDonald et al,^[20]^ RNVG in the study by Jones et al,^[19]^ and echocardiography in the study by Hunter et al.^[18]^ Additionally, if the RNVG was chosen, the LVEF improvement remained significant with an MD change to 7.81%.

One study suggested that ejection fraction measurements with these 3 techniques are not interchangeable and that echocardiography exhibits the greatest variation. Thus, CMR is the preferred technique for LVEF estimation in HF patients.^[21]^ However, CMR images come from several cardiac cycles, whereas those of RNVG are created from 20 minutes’ worth of heartbeats, which may make RNVG more reliable particularly for HF patients with AF. Thus, whether the results would have changed if only RNVG was applied for LVEF measurement remains to be determined and the optimal estimation technique for HF patients with AF also remains uncertain.

We also found that although the LVEF improvement in the catheter ablation arm failed to reach significance in Jones study, its extent (10.9%) was greater than that in Hunter (8.1%) and MacDonald studies (4.5%). The rate control groups of the latter 2 studies exhibited small LVEF improvements or even declines.

One potential possibility is that the participants were not adequately or even poorly rate controlled in these 2 studies because only Jones study reported well-controlled heart rates,^[19]^ which may have resulted in the inferiority of the medical rate control therapy. Moreover, all 3 studies administered beta blockers as the main rate control therapy, and some studies have failed to demonstrate the benefits of beta blockers.^[9]^

However, even compared with strict rate control therapy, as observed in another study, PABA CHF, which compared atrioventricular node ablation plus biventricular pacing with catheter ablation (rhythm control) for AF patients with HF, catheter ablation still elicited significantly improved LV function.^[13]^

Catheter ablation also resulted in significantly improved cardiac function and exercise capacity including the NYHA class, peak VO_2_, and QOL compared with medical rate control therapy.

The peak VO_2_ is thought to be a strong prognostic indicator because it can reflect the exercise tolerance degrees of HF patients.^[22]^ Moreover, the improvements in the peak VO_2_ in the ablation group were greater than those of other HF studies in which medication or cardiac resynchronization (CRT) were applied, which suggests an improvement in better prognosis due to the restoration of the sinus rhythm via catheter ablation.

The current recommendations indicate that only patients with symptomatic AF refractory to medical treatment should obtain catheter ablation therapy. In our study, because symptomatic AF patients were not included, catheter ablation may, to a large extent, be considered as the treatment for HF rather than for AF.

Both BNP and NT-proBNP have recently been research hotspots. Our analysis revealed nonsignificant trends toward decreases of both in the ablation group. BNP and NT-proBNP have been further examined and determined to have diagnostic and prognostic value for HF, and therapy guided by BNP may improve HF outcomes.^[23]^ As the number of studies were limited, the explanation for this result may be underpowered. Additional studies are needed to further explain the roles of BNP and NT-proBNP in AF and HF patients.

The included studies also assessed the QOLs with the MLHFQ, SF-36, or KCCQ, and the majority demonstrated improvements in the patients who underwent ablation, especially in terms of the MLHFQ and SF-36 physical aspects, and these improvements led to both better life quality and favorable prognostic outcomes.

All these changes in the ablation group were vital because they were not only associated with improved cardiac function, exercise capacity, and symptoms, but also may have predicted favorable prognoses, reductions in hospitalization, and even reductions in mortality.

Compared with medical therapy, catheter ablation in AF is inevitably associated with significant risks because it is an invasive procedure. However, it is reassuring that catheter ablation is able to restore and maintain the sinus rhythms of AF and HF patients with a relatively low risk of complications and side effects. Among the analyzed studies, the procedural complication rates ranged from 7.7% to 15.4%. These rates are consistent with results of a recent meta-analysis that aimed to examine the efficiency and safety of catheter ablation for AF and HF patients.^[6]^ However, the limited number of events and the overall sample size should be taken into account.

In the ablation arm, the percentages of patients who remained in sinus rhythm were 50%,^[20]^ 81%,^[18]^ and 88%^[19]^ during the follow-up. However, the lengths of follow-up were relatively short in the included studies. The longest duration was 12 months, and studies with longer follow-up durations (mean of 46.2 months) have reported high AF recurrence rates and the requirement of repeated procedures to achieve sinus rhythm maintenance.^[24,25]^

Considering that a portion of the patients in the ablation group may have required repeated procedures in the subsequent days, the risk-benefit ratio for catheter ablation may be altered in comparison with medical rate therapy. Thus, the long-term superiority of catheter ablation for persistent AF patients with HF is uncertain.

Moreover, for those AF and HF patients with preserved LVEF, whether catheter ablation still has benefits compared with rate control also remains to be determined despite that some single-arm studies that have demonstrated its efficiency and safety.^[26]^ Further trials are also needed to determine whether ablation therapy can improve hard end points such as mortality for AF and HF patients.

Several limitations to our study should be noted. First, the numbers of included studies and patients were relatively small, and only 3 RCTs were included. These studies were pooled due to the limited number. Second, our study depended only on the data reported in studies, some end point data were unavailable, and considering the limited number of studies, publication and reporting biases were inevitable to some extent. Third, follow-up lengths were short; the longest was 12 months, which may have been insufficient for complications and recurrence to occur. Thus, the analysis of the long-term results was underpowered.

## 5 Conclusion

In this comprehensive review and meta-analysis of 3 RCTs that included 143 patients, catheter ablation was found to significantly improve LVEF, NYHA class, peak VO_2_, and QOL in persistent AF patients with HF compared with medical rate control therapy. A relatively low complication rate was observed in the ablation-treated patients. Certain limitations, including the limited numbers of studies and patients and short-term follow ups, should be considered.

## Acknowledgments

The authors thank the National Natural Science Foundation of China (81170270) for the support.

## References

[1] Caldeira D David C Sampaio C Rate vs rhythm control in patients with atrial fibrillation and heart failure: a systematic review and meta-analysis of randomised controlled trials. *Eur J Intern Med* 2011; 22:448–455. 21925051 10.1016/j.ejim.2011.05.001

[2] Chatterjee S Sardar P Lichstein E Pharmacologic rate versus rhythm-control strategies in atrial fibrillation: an updated comprehensive review and meta-analysis. *PACE* 2013; 36:122–133. 22978656 10.1111/j.1540-8159.2012.03513.x

[3] Blandino A Toso E Scaglione M Long-term efficacy and safety of two different rhythm control strategies in elderly patients with symptomatic persistent atrial fibrillation. *J Cardiovasc Electrophysiol* 2013; 24:731–738. 23551460 10.1111/jce.12126

[4] Kim KH Na JO Nam GB Effect of catheter ablation on the left ventricular mass index and other echocardiograph parameters in atrial fibrillation patients: Comparison with antiarrhythmic drug treatment. *J Echocardiogr* 2011; 9:51–58. 27276880 10.1007/s12574-010-0069-2

[5] Reynolds MR Gunnarsson CL Hunter TD Health outcomes with catheter ablation or antiarrhythmic drug therapy in atrial fibrillation: results of a propensity-matched analysis. *Circ Cardiovasc Qual Outcomes* 2012; 5:171–181. 22373904 10.1161/CIRCOUTCOMES.111.963108

[6] Dagres N Varounis C Gaspar T Catheter ablation for atrial fibrillation in patients with left ventricular systolic dysfunction. A systematic review and meta-analysis. *J Card Fail* 2011; 17:964–970. 22041335 10.1016/j.cardfail.2011.07.009

[7] Rienstra M Van Gelder IC Ventricular rate control of atrial fibrillation in heart failure. *Heart Fail Clin* 2013; 9:397–406. 24054473 10.1016/j.hfc.2013.07.004

[8] Cullington D Goode KM Zhang J Is heart rate important for patients with heart failure in atrial fibrillation? *JACC Heart Fail* 2014; 2:213–220. 24952686 10.1016/j.jchf.2014.01.005

[9] Kotecha D Holmes J Krum H Efficacy of beta blockers in patients with heart failure plus atrial fibrillation: an individual-patient data meta-analysis. *Lancet* 2014; 384:2235–2243. 25193873 10.1016/S0140-6736(14)61373-8

[10] Chen MS Marrouche NF Khaykin Y Pulmonary vein isolation for the treatment of atrial fibrillation in patients with impaired systolic function. *J Am Coll Cardiol* 2004; 43:1004–1009. 15028358 10.1016/j.jacc.2003.09.056

[11] Gentlesk PJ Sauer WH Gerstenfeld EP Reversal of left ventricular dysfunction following ablation of atrial fibrillation. *J Cardiovasc Electrphysiol* 2007; 18:9–14. 10.1111/j.1540-8167.2006.00653.x 17081210

[12] Hsu LF Jais P Sanders P Catheter ablation for atrial fibrillation in congestive heart failure. *N Engl J Med* 2004; 351:2373–2383. 15575053 10.1056/NEJMoa041018

[13] Khan MN Jais P Cummings J Pulmonary-vein isolation for atrial fibrillation in patients with heart failure. *N Engl J Med* 2008; 359:1778–1785. 18946063 10.1056/NEJMoa0708234

[14] Lutomsky BA Rostock T Koops A Catheter ablation of paroxysmal atrial fibrillation improves cardiac function: a prospective study on the impact of atrial fibrillation ablation on left ventricular function assessed by magnetic resonance imaging. *Europace* 2008; 10:593–599. 18385123 10.1093/europace/eun076

[15] Tondo C Mantica M Russo G Pulmonary vein vestibule ablation for the control of atrial fibrillation in patients with impaired left ventricular function. *PACE* 2006; 29:962–970. 16981920 10.1111/j.1540-8159.2006.00471.x

[16] Moher D Liberati A Tetzlaff J Preferred reporting items for systematic reviews and meta-analyses: the PRISMA statement. *Int J Surg* 2010; 8:336–341. 20171303 10.1016/j.ijsu.2010.02.007

[17] Higgins J Green S Cochrane Collaboration. Cochrane Handbook for Systematic Reviews of Interventions. Hoboken, NJ: John Wiley & Sons; 2008.

[18] Hunter RJ Berriman TJ Diab I A randomized controlled trial of catheter ablation versus medical treatment of atrial fibrillation in heart failure (the CAMTAF trial). *Circ Arrhythm Electrophysiol* 2014; 7:31–38. 24382410 10.1161/CIRCEP.113.000806

[19] Jones DG Haldar SK Hussain W A randomized trial to assess catheter ablation versus rate control in the management of persistent atrial fibrillation in heart failure. *J Am Coll Cardiol* 2013; 61:1894–1903. 23500267 10.1016/j.jacc.2013.01.069

[20] MacDonald MR Connelly DT Hawkins NM Radiofrequency ablation for persistent atrial fibrillation in patients with advanced heart failure and severe left ventricular systolic dysfunction: a randomised controlled trial. *Heart* 2011; 97:740–747. 21051458 10.1136/hrt.2010.207340

[21] Bellenger NG Burgess MI Ray SG Comparison of left ventricular ejection fraction and volumes in heart failure by echocardiography, radionuclide ventriculography and cardiovascular magnetic resonance; are they interchangeable? *Eur Heart J* 2000; 21:1387–1396. 10952828 10.1053/euhj.2000.2011

[22] Chase PJ Kenjale A Cahalin LP Effects of respiratory exchange ratio on the prognostic value of peak oxygen consumption and ventilatory efficiency in patients with systolic heart failure. *JACC Heart Fail* 2013; 1:427–432. 24621975 10.1016/j.jchf.2013.05.008 PMC7296992

[23] Xin W Lin Z Mi S Does B-type natriuretic peptide-guided therapy improve outcomes in patients with chronic heart failure? A systematic review and meta-analysis of randomized controlled trials. *Heart Fail Rev* 2015; 20:69–80. 24888383 10.1007/s10741-014-9437-8

[24] Bortone A Pujadas-Berthault P Karam N Catheter ablation in selected patients with depressed left ventricular ejection fraction and persistent atrial fibrillation unresponsive to current cardioversion. *Europace* 2013; 15:1574–1580. 23585251 10.1093/europace/eut088

[25] Anselmino M Grossi S Scaglione M Long-term results of transcatheter atrial fibrillation ablation in patients with impaired left ventricular systolic function. *J Cardiovasc Electrophysiol* 2013; 24:24–32. 23140485 10.1111/j.1540-8167.2012.02419.x

[26] Machino-Ohtsuka T Seo Y Ishizu T Efficacy, safety, and outcomes of catheter ablation of atrial fibrillation in patients with heart failure with preserved ejection fraction. *J Am Coll Cardiol* 2013; 62:1857–1865. 23916940 10.1016/j.jacc.2013.07.020

